# Supplementary material for: Tracking putative Microcystis viruses and virus-host associations across distinct phases of a Microcystis-dominated bloom
Source: mSystems. 2025 Sep 22;10(10):e00575-25. doi: 10.1128/msystems.00575-25 (PMC12542629; doi:10.1128/msystems.00575-25)
Supplement: Supplemental Figures — Figures S1 to S9. [file msystems.00575-25-s0001.pdf]

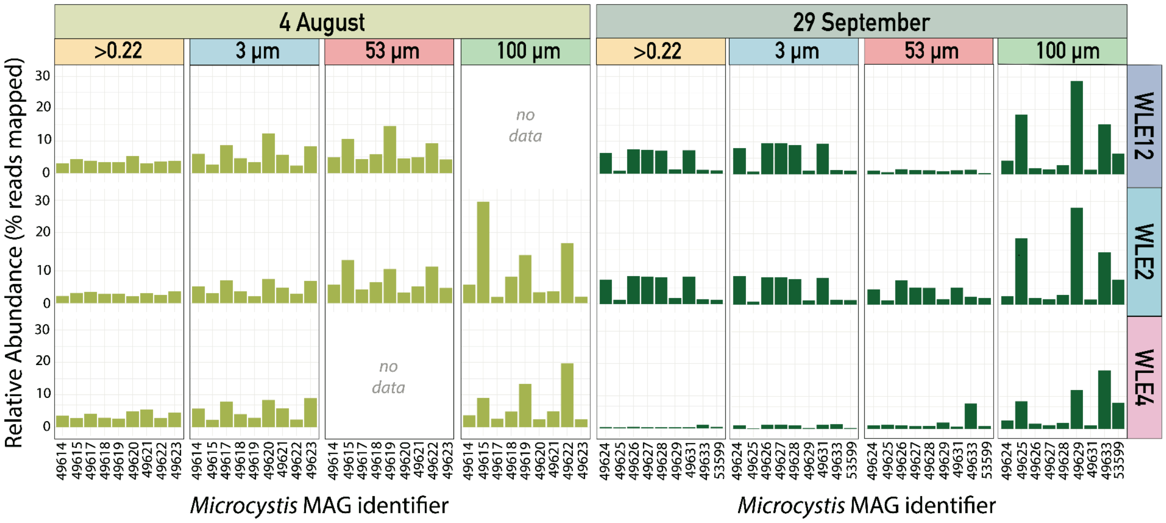

Supplementary Figure 1. Relative abundances of 17 *Microcystis* MAGs found on the bloom peak dates. 9 MAGs were identified on August 4th and 8 MAGs on September 29th. Relative abundances are based on the fraction of reads from each sample that is mapped to each MAG in a competitive read mapping to all assembled MAGs from that sample. No data exist for 4 Aug 100- µm at WLE12 and 53 µm at WLE4.

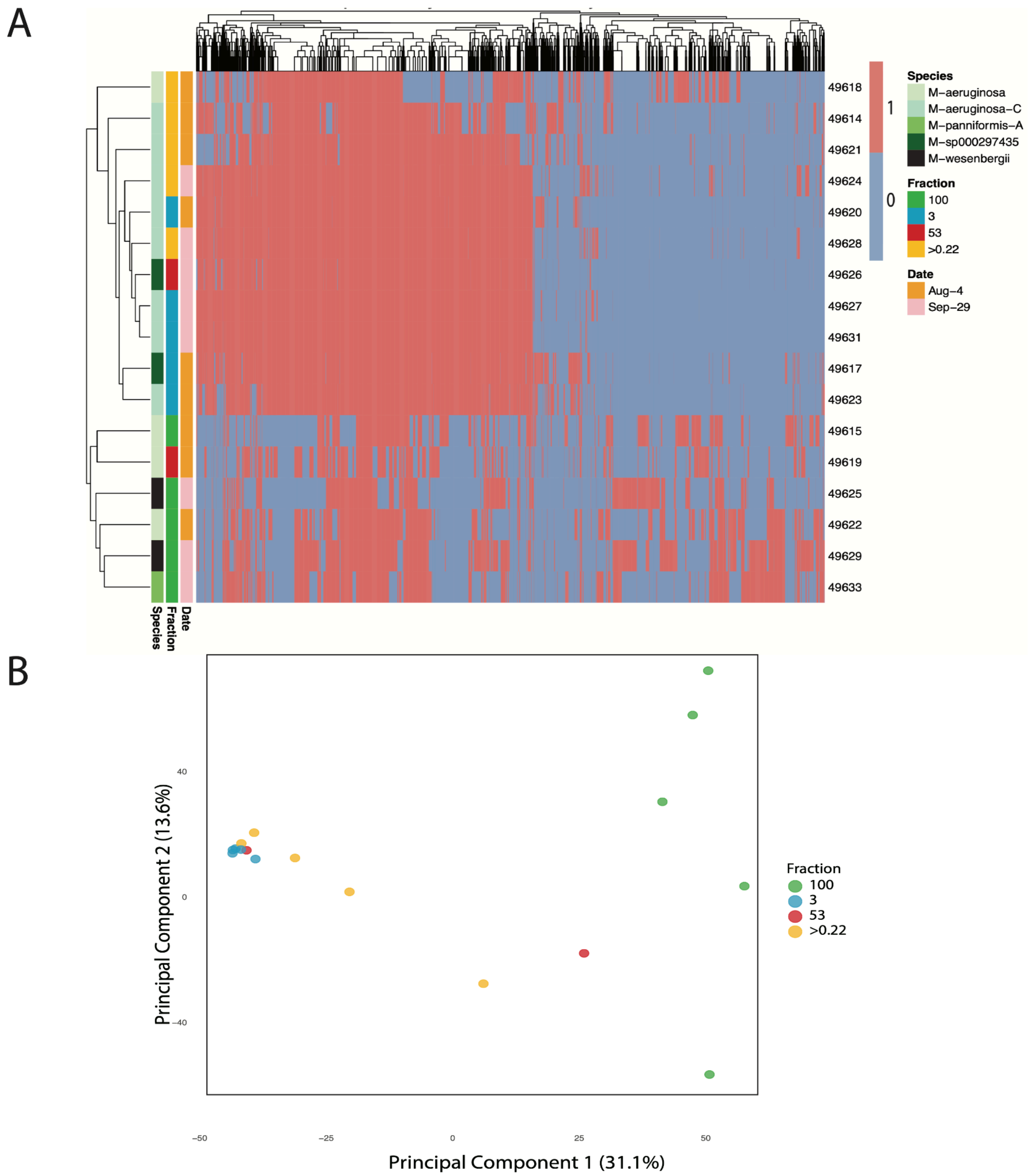

Supplementary Figure 2. Gene cluster analysis of *Microcystis* strains identified at 2014 cHAB peaks. (A) Heatmap of *Microcystis* pangenome gene clusters grouped by *Microcystis* strain and *Microcystis* pangenome gene cluster. Red cells indicate pangenome gene cluster presence, while blue cells indicate pangenome gene cluster absence. (B) PCA of *Microcystis* pangenome gene clusters by *Microcystis* MAG. Point color indicates which size fraction a given *Microcystis* MAG belongs to.

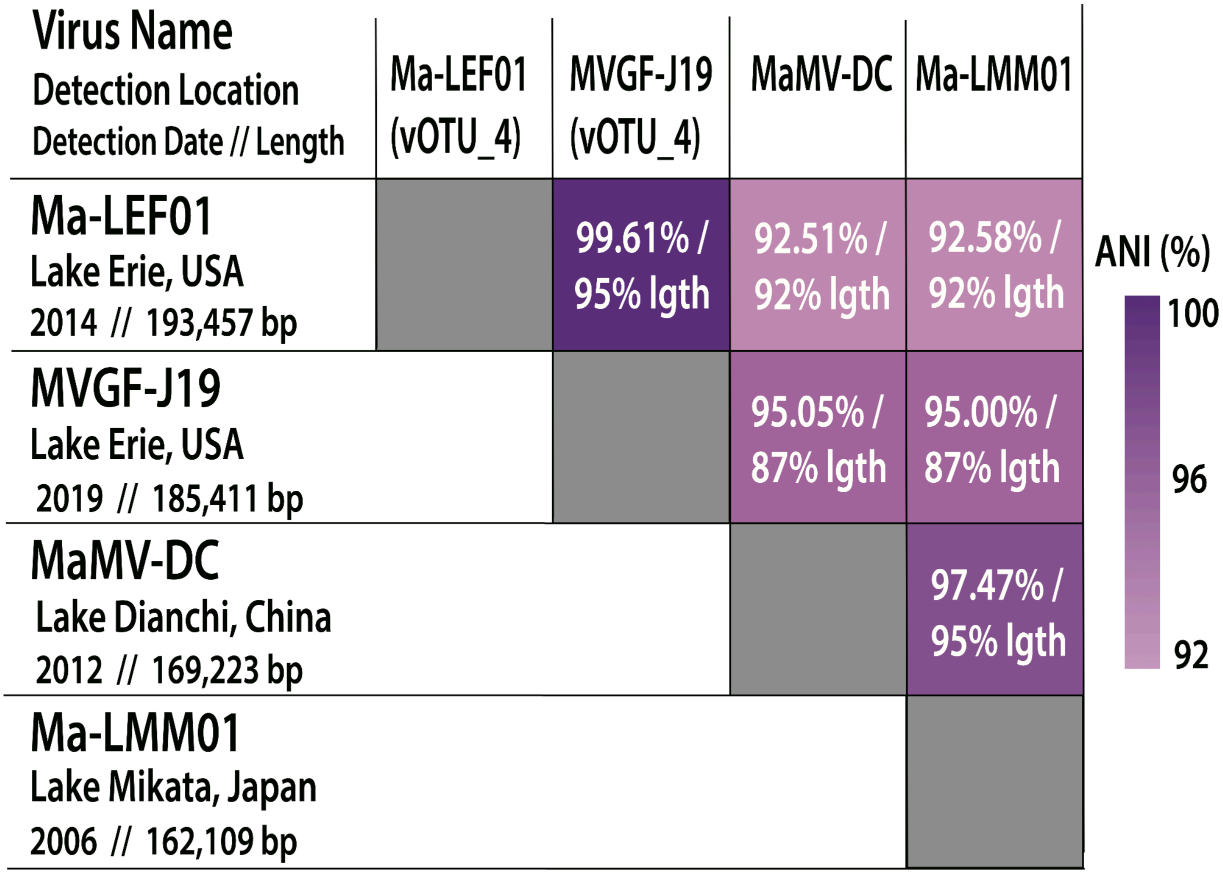

Supplementary Figure 3. Pairwise genome similarity between the Lake Erie Ma-LEF01 and three closely related known Microcystis viruses (Ma-LMM01 and MaMV-DC) and viral contig (MVGF-J19). Similarity reported as average nucleotide identity (%) and alignment fraction (%) relative to the shortest of the two being compared.

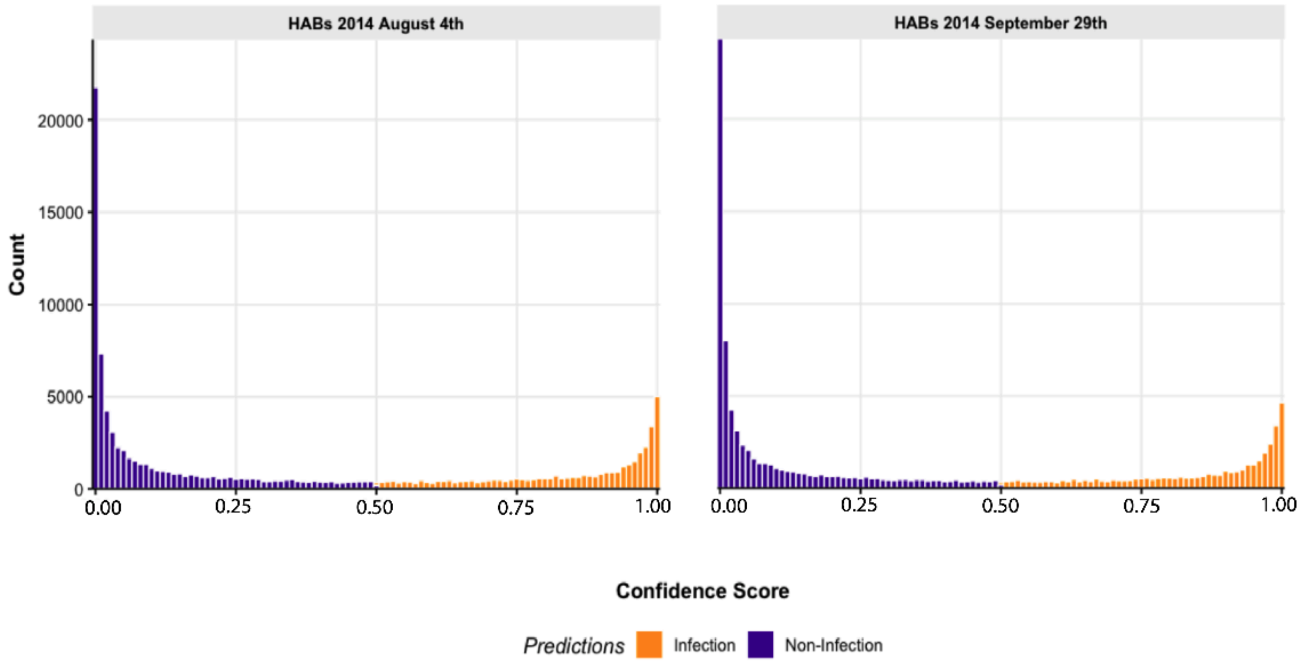

Supplementary Figure 4. Score distribution from VHIP for each prediction made on August 4th and September 29th. A score of 0 indicates a strong non-infection prediction whereas a score of 1 indicates a strong infection prediction, with values in between representing varying degrees in the confidence in the prediction.

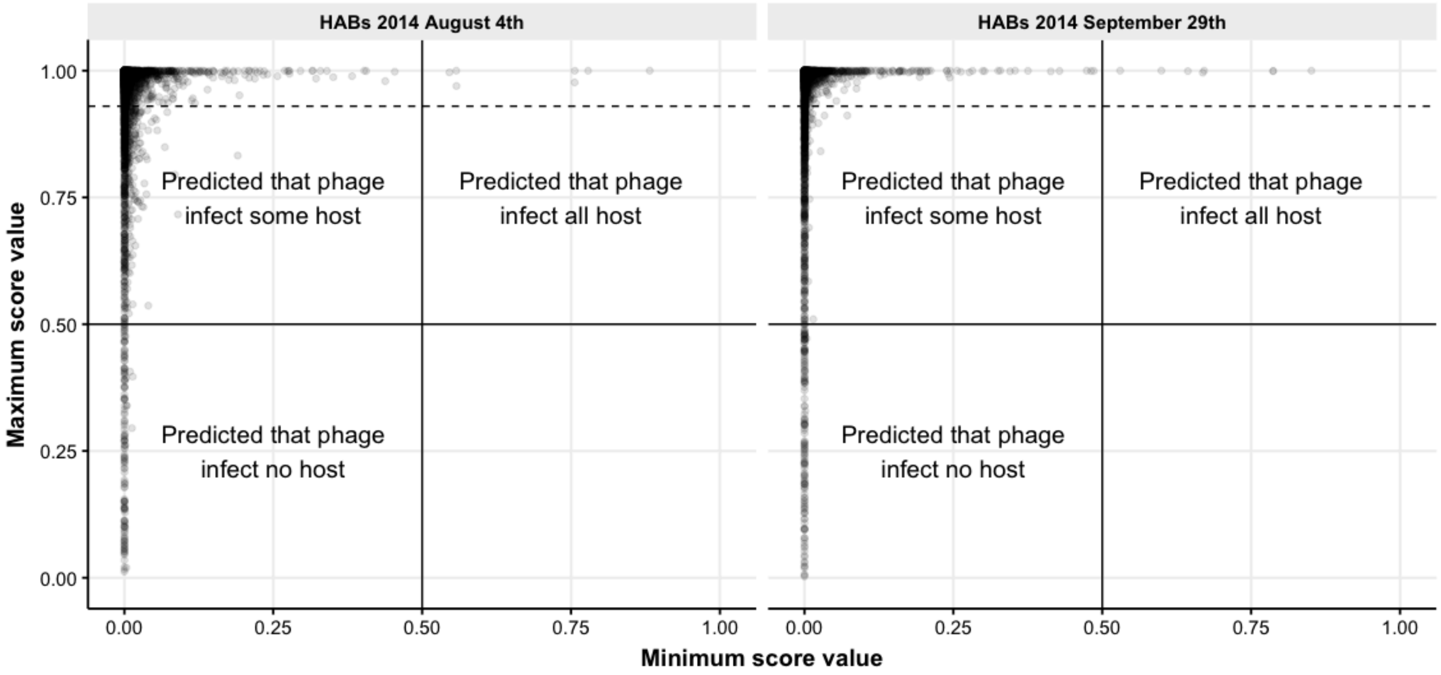

Supplementary Figure 5. Plotting the minimum score and the maximum score from VHIP's confidence score for each virus.

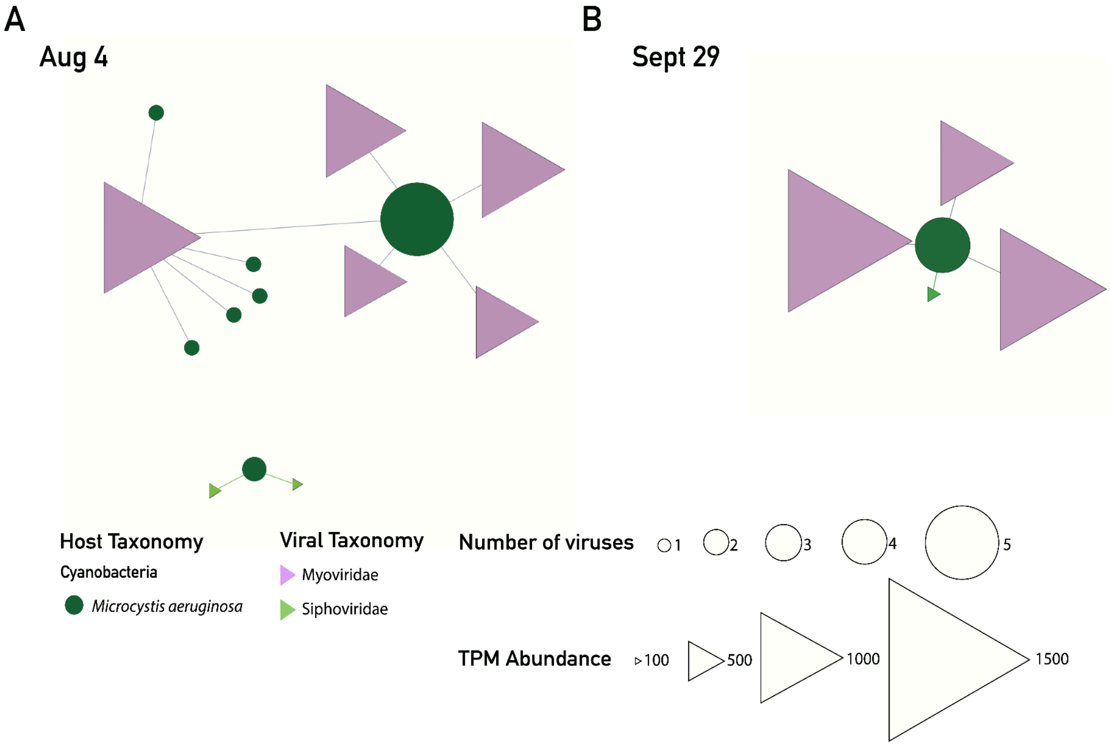

Supplementary Figure 6. Networks of predicted infections based on CRISPR spacer matches between *Microcystis* viruses (>10 kb viral contigs) and bacterial host MAGs identified on the 4 Aug and 29 Sept bloom metagenomes. (A) Predicted spacer infection network of 4 August toxic bloom peak. (B) Predicted spacer infection network of 29 September non-toxic bloom peak. Circle nodes are host MAGs; circle size represents the number of viruses predicted to infect a given host. Triangle nodes are viruses. Node size represents TPM abundance. Node colors represent assigned taxonomy.

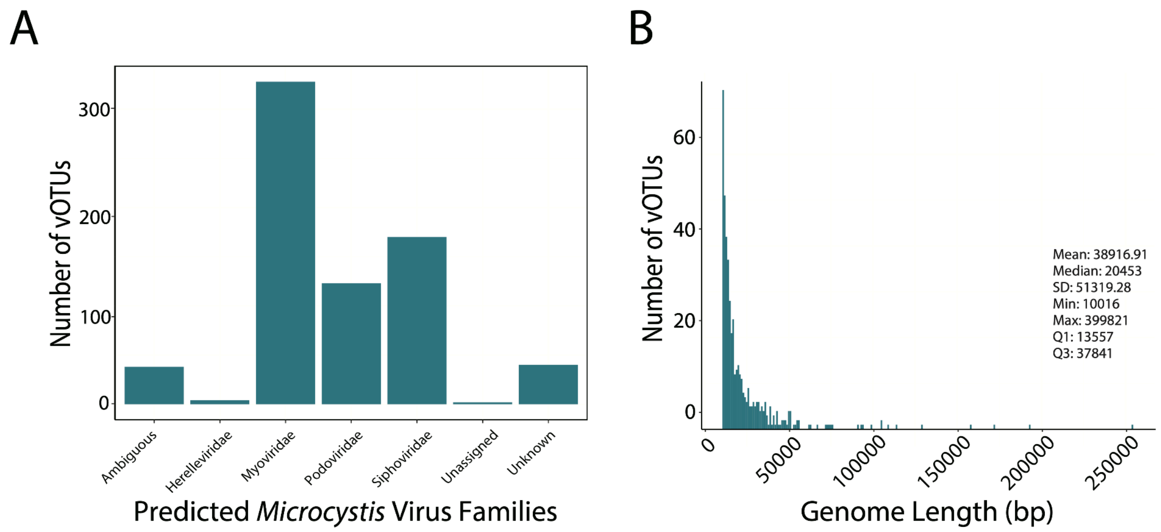

Supplementary Figure 7. Summary of predicted *Microcystis* vOTUs in Lake Erie. (A) Overview of taxonomic assignment by family for predicted *Microcystis* vOTUs. (B) Length distribution of predicted *Microcystis* vOTUs. Predicted *Microcystis* vOTUs shown here are the result of a 10kb sequence length minimum applied and a >93% probability of infection.

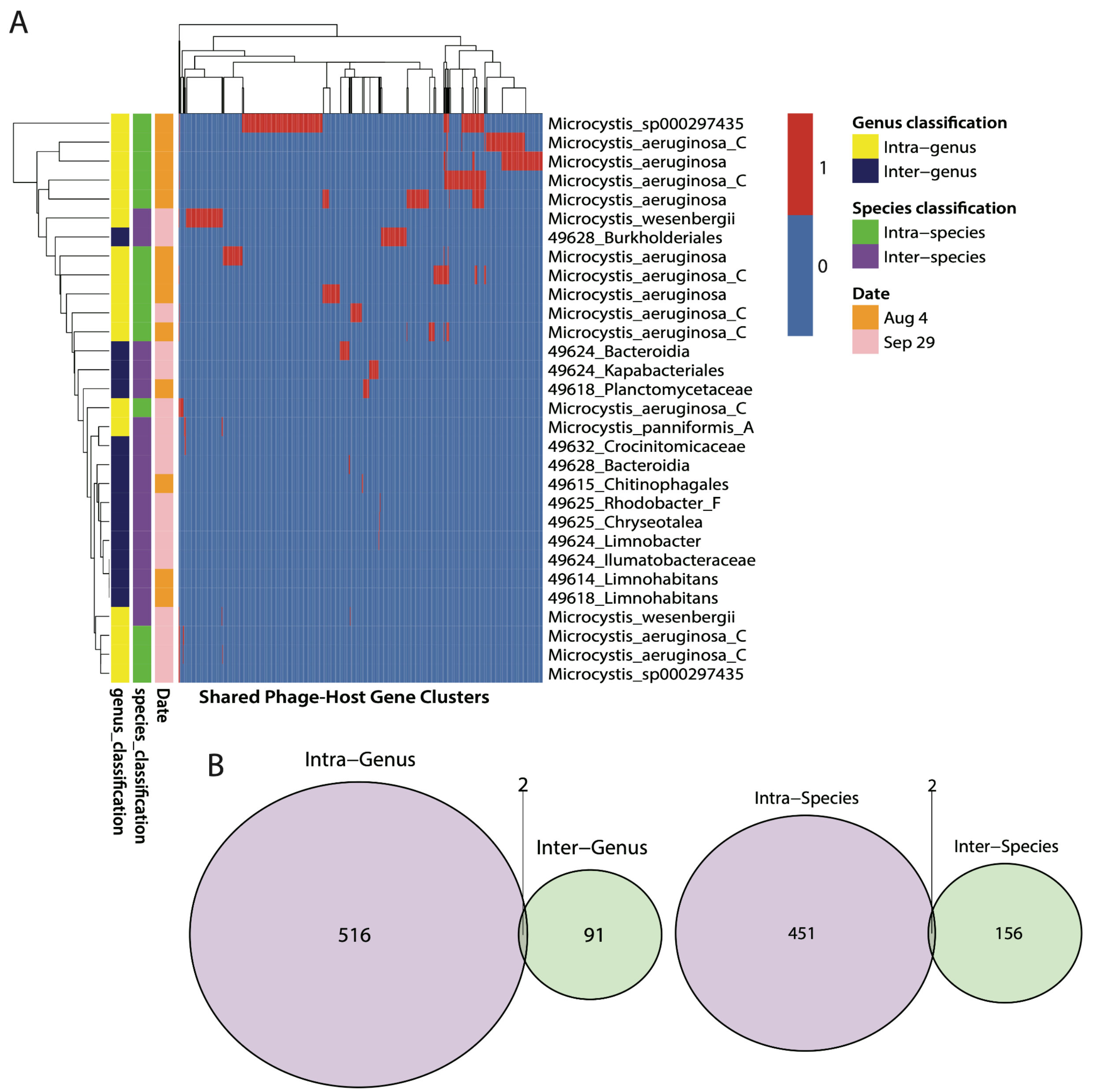

Supplementary Figure 8. Gene cluster analysis of shared genes between putative *Microcystis* phage and bloom peak hosts. (A) Heatmap of shared phage-host gene clusters grouped by host and shared phage-host gene cluster. Red cells indicate gene cluster presence, while blue cells indicate gene cluster absence. (B) Venn diagrams of shared phage-host gene clusters at both the inter/intra genus and species levels.

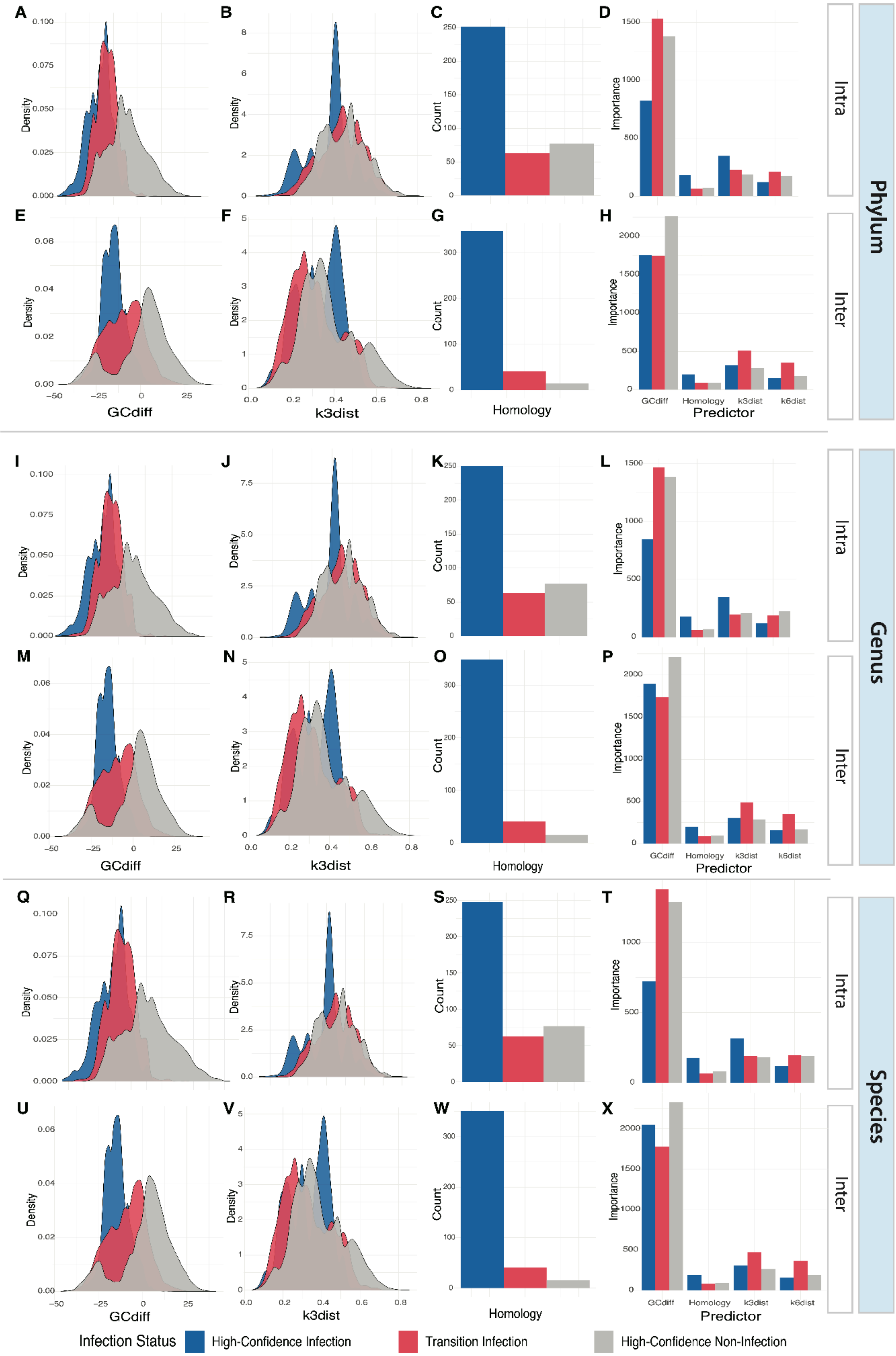

A
B
C
D
E
F
G
H
I
J
K
L
M
N
O
P
Q
R
S
T
U
V
W
X
Density
Count
Importance
GCdiff
k3dist
Homology
Predictor
GCdiff
Homology
k3dist
k6dist
Intra
Inter
Phylum
Genus
Species
Infection Status
High-Confidence Infection
Transition Infection
High-Confidence Non-Infection

Supplementary Figure 9. Analysis of inter-phyla phage-host predictions with VHIP. (A, E, I, M, Q, U) Density plot of GCdiff values for different infection statuses, with points colored according to infection status: high-confidence infection (blue), transition infection (red), and high-confidence non-infection (gray). (B, F, J, N, R, V) Density plot of k3dist values across infection statuses. (C, G, K, O, S, W) Bar plot showing the count of predictions without a shared sequence between host and phage (Homology = 1) across the three infection statuses. (D, H, L, P, T, X) Variable importance across VHIP model predictors (GCdiff, Homology, k3dist, and k6dist), comparing their significance for each infection status group. Feature importance analysis for virus-host infection predictions across taxonomic levels (phylum, genus, species) reveals distinct genomic patterns. GCdiff emerged as the strongest predictor of infection across all levels, with inter-taxon predictions showing greater reliance on GCdiff than intra-taxon predictions. High-confidence infections (infection probability ≥0.93) were associated with GCdiff values between -15 and -5, while high-confidence non-infections (≤0.12) clustered between 5 and 10. Transition infections overlapped with high-confidence infections in intra-taxon predictions but displayed distinct peaks in inter-taxon predictions. High-confidence infections were 5-7 times more likely to share sequences with hosts. k-mer frequency analyses (k3 and k6) showed substantial overlap across infection categories, limiting their predictive power. These results support a hierarchical model of virus-host coevolution, where different genomic features influence infection predictions at varying taxonomic levels.
